# Assessment of Density Variations of Marine Sediments with Ocean and Sediment Depths

**DOI:** 10.1155/2014/823296

**Published:** 2014-03-06

**Authors:** R. Tenzer, V. Gladkikh

**Affiliations:** ^1^Institute of Geodesy and Geophysics, School of Geodesy and Geomatics, Wuhan University, 129 Luoyu Road, Wuhan 430079, China; ^2^International IT University, 34A/8A Manas/Zhandosov Street (Intersection), Almaty 050040, Kazakhstan

## Abstract

We analyze the density distribution of marine sediments using density samples taken from 716 drill sites of the Deep Sea Drilling Project (DSDP). The samples taken within the upper stratigraphic layer exhibit a prevailing trend of the decreasing density with the increasing ocean depth (at a rate of −0.05 g/cm^3^ per 1 km). Our results confirm findings of published studies that the density nonlinearly increases with the increasing sediment depth due to compaction. We further establish a 3D density model of marine sediments and propose theoretical models of the ocean-sediment and sediment-bedrock density contrasts. The sediment density-depth equation approximates density samples with an average uncertainty of about 10% and better represents the density distribution especially at deeper sections of basin sediments than a uniform density model. The analysis of DSDP density data also reveals that the average density of marine sediments is 1.70 g/cm^3^ and the average density of the ocean bedrock is 2.9 g/cm^3^.

## 1. Introduction

The understanding of a structural density composition of marine sediments plays an important role in several multidisciplinary research areas. In marine gravimetric studies, sediment corrections have been often calculated by adopting a uniform density distribution, irrespective of physical properties and mineral composition of marine sediments. Some authors developed more complex density-depth equations based on the analysis of specific sediment types and their physical properties. The gross density structure and thickness of basin sediments were obtained from either ocean drilling data or seismic surveys. These data were incorporated in gravimetric marine (or coastal) profile models by Donato and Tully [1], Dimitropoulos and Donato [2], Foucher et al. [3], Zervos [4], Holliger and Klemperer [5], Thorne and Watts [6], and others. The sediment density models are also required in modeling and subsequent removing of the crustal loading caused by sedimentary accumulations. Examples of the applied sediment isostatic compensation include, but are not limited to, studies of paleobathymetry (e.g., [7–12]), evolution of sedimentary basins (e.g., [13–17]), thermal structure of the oceanic lithosphere (e.g., [18]), continental shelf basins (e.g., [19, 20]), and historical sea level (e.g., [21]).

The uniform density distribution has been often assumed in gravimetric studies of marine sediments. Estimates of the average sediment density can be found, for instance, in Sclater et al. [22–24], Crough [25], Renkin and Sclater [9], Hayes [8], Kane and Hayes [11], and Coffin [26]. These authors provided the average density estimates between 1.7 and 1.95 g/cm^3^; for summary, see also Sykes [27]. Large density variations within marine sediment basins depend on their physical properties and mineral composition (cf. [28–32]). Several authors developed and applied more complex density models taking into consideration a particular sediment type and its specific physical properties (e.g., porosity and compaction). Hamilton and Menard [33] studied the density and porosity of sea-floor sediments. Hamilton [34] investigated how the density and porosity of deep sea sediments vary with depth. His study focused on four types of sediments, namely, calcareous and siliceous oozes, pelagic clay, and terrigenous sediments. He then derived a regression function for each sediment type, but he emphasized that these density models should not be used for sediment depths greater than those indicated in his tables and figures (500 m for calcareous ooze, 250 m for radiolarian ooze, 300 m for pelagic clay, 500 m for diatomaceous ooze, and 1300 m for terrigenous sediments). He also stated that there is no universal curve of density or porosity with depth in sediments or rocks even if separated into various sediment types. Granser [35] applied an exponential density-depth function for a gravimetric interpretation of sedimentary basins. Mienert and Schultheiss [36] compared physical properties of marine sediments at two (near coastal) drill sites in oceanic high and low biogenic productivity zones. They concluded that increased biogenic silica concentrations in sediments beneath the upwelling area cause a low average grain density (<2.4 g/cm^3^), low wet-bulk density (<1.6 g/cm^3^), and low shear strength (<60 kPa). The sediments present in the nonupwelling area were found to have higher average carbonate concentrations (40–90%) and reflect a steady increase in wet-bulk density (decrease in porosity) with depth (0.12 g/cm^3^ per 100 m). Cowie and Karner [37] established an exponential function of porosity to describe the depth-dependent density change due to compaction. They used the regional sediment data from the North Sea and the Rhine Graben. Sykes [27] calculated the isostatic correction for the sediment load on oceanic basements, using a uniform density structure. He then applied an alternative method in which the sediment density was calculated based on applying Hamilton's [34] depth-dependent equations for calcareous, clay, and terrigenous sediment sequences. This alternative method takes into consideration the ocean depth and the total sediment thickness. For example, deep ocean sediments (>4 km) are typically formed from clay, and thick sediment sequences (>2 km) usually have a high terrigenous component, while shallow sequences are likely to be calcareous rich. He compared the density estimates of both theoretical models with measurements taken from 10 drill sites and from densities derived from seismic interval velocities. He demonstrated that Hamilton's [34] equations produced isostatic corrections similar to those derived by published methods for sediment sequences less than 1 km, but compensated better for thicker sediment deposits. Wang et al. [38] applied the stepwise linearly approximated density-depth function of Cowie and Karner [37] to estimate the crust thickness anomalies in the North Atlantic Ocean basin, using gravimetric methods. Accurate sediment density models are also required in continental studies. Artemjev et al. [39], for instance, applied a depth-dependent sediment density model in the gravimetric study of subcrustal density inhomogeneities of the Northern Eurasia.

In this study, we derive the sediment density-depth equations that define the density distribution as a function of the ocean and sediment depths. Since global datasets of the ocean depths and the total sediment thickness are currently provided with a relatively high resolution, the 3D density model of marine sediments can be readily applied in gravimetric marine studies. The application of more specific density distribution models in global studies is currently restricted by the fact that the geographical distribution of marine sediment types (and their physical properties) is poorly documented. Moreover, the information on sediment type and its physical properties is not provided in datasets used for the analysis in this study.

## 2. Marine Sediment Data

The gravimetric forward modeling of marine sediment structures requires accurate data of the ocean depth, the total sediment thickness, and the sediment density distribution. The global bathymetric models currently available are provided with a relatively high resolution. The National Geophysical Data Center (NGDC) of the U.S. National Oceanic and Atmospheric Administration (NOAA) contains a 1 arc min global model ETOPO1 that integrates land topography and ocean bathymetry [40]. The NGDC includes a 5 arc min data of the total sediment thickness for the world's oceans and marginal seas [41]. This database was compiled using previously published isopach maps [42–46], ocean drilling results, and seismic reflection profiles archived in the NGDC as well as seismic data and isopach maps available as a part of the International Geological-Geophysical Atlas of the Pacific Ocean [47]. We note that the NGDC data of the total sediment thickness are not provided in the Arctic Ocean and some other parts of the oceans. The NGDC database also contains density files of marine sediments. These records were prepared from core data collected during the Deep Sea Drilling Project (DSDP) produced in 2000 by the U.S. Department of Commerce, NOAA, National Environmental Satellite, Data, and Information Service, National Geophysical Data Center, and collocated World Data Center for Marine Geology and Geophysics, Boulder. These records were prepared in cooperation with the U.S. Science Support Program, the Joint Oceanographic Institutions Inc., and the U.S. National Science Foundation. The DSDP was an international study of the global oceans supported by the U.S. National Science Foundation and the governments of the former Federal Republic of Germany, France, Japan, the United Kingdom, and the former Soviet Union. The data were collected and compiled within the DSDP framework from 1968 through to 1987 under the auspices of the Scripps Institution of Oceanography, the science operator of the DSDP. We note that core data from the Ocean Drilling Program, also included in the DSDP database, were not used due to existing errors and inconsistencies.

The density water content, porosity, density, and grain density of marine sediments were measured aboard the Glomar Challenger (which is deep sea research and scientific drilling vessel for oceanography and marine geology studies) on core samples. Several different techniques were applied to measure the sediment density such as syringe, chunk, and cylinder techniques [48, 49]. In this study, we used the wet-bulk density measurements, including the ocean depth of drill sites and drilling depths of the taken density samples. It is worth mentioning that the error estimates of the NGDC measurements were not specified. The most significant factor affecting the accuracy of density measurements is likely changing physical conditions of the measured core samples aboard compared to their deposit location.

**Figure 1:**
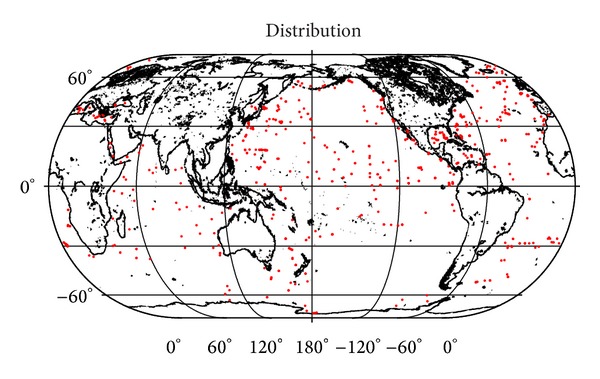
Location map of the 716 DSDP drill sites.

The DSDP files contain total of 21937 density samples collected at 716 drilling sites (as retrieved from the DSDP database at 20/05/2013). We note here that the DSDP database contains also additional 49 files, which have the density column empty. Majority of these drill sites are located in the northern hemisphere, while there is no sufficient cover in large parts of the southern hemisphere and almost total absence of drilling sites in the Arctic Ocean (see Figure 1).

## 3. Data Acquisition

**Figure 2:**
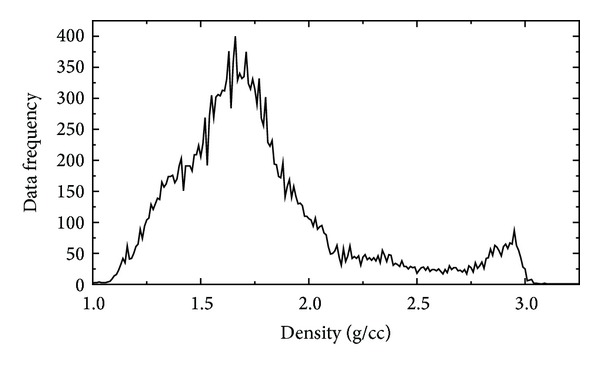
Histogram of the DSDP density samples.

The DSDP densities range between 0.95 and 4.42 g/cm^3^. As seen from the histogram in Figure 2, the density distribution exhibits two distinctive picks, with two local maxima roughly at 1.7 and 2.9 g/cm^3^. The DSDP files thus comprise not only sediment but also bedrock density samples. Since the analysis here is limited to marine sediments, we separated sediments from bedrock samples using the selection criterion based on the assumption that the sediment density should not exceed 2.60 g/cm^3^. This selection criterion is verified in Section 5. The 20347 selected samples have densities between 0.95 and 2.60 g/cm^3^, with a mean of 1.70 g/cm^3^and a standard deviation of 0.29 g/cm^3^. The ocean depths of the DSDP drill sites range from 193 m to 7034 m. The maximum drilling depth of these samples is at 1737 m. It is thus worth mentioning that density models developed in this study are applicable to a maximum ocean depth of 7 km and a maximum sediment thickness of 1.7 km. Their validity beyond these limits has to be further verified when data become available.

**Figure 3:**
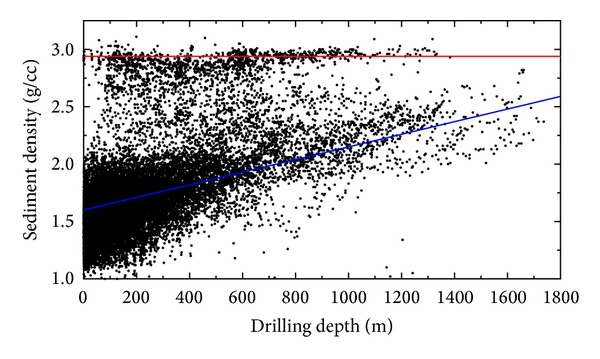
Scatter plot of the DSDP sediment density samples with respect to the drilling depths. The linear regression functions were used to approximate density trends within marine sediments (lower line) and bedrock samples (upper line). The chosen maximum sediment density is 2.60 g/cm^3^.

We applied a linear regression model to examine density changes within sediment and bedrock samples selected according to a criterion that the maximum sediment density is 2.60 g/cm^3^. The linear regression trends, which approximate the density distributions within the marine sediments and the ocean bedrock, are shown in Figure 3. The sediments exhibited the expected trend of a depth-increasing density. In contrast, the bedrock densities are without an apparent systematic trend.

## 4. Numerical Analysis and Results

The selected DSDP density samples were used to analyze a density change with respect to ocean and sediment depths. In both cases, we applied functional relations for describing the actual density distribution, which can be readily used in gravimetric methods based on solving Newton's volumetric integral.

### 4.1. Lateral Density Variation

We first inspected a lateral density variation within the upper sediment layer. For this purpose, we selected the shallowest density sample at each site for drilling depths no greater than 50 m. This selection criterion is verified in Section 5. We then applied the least-squares analysis to fit a linear regression function to the selected 457 DSDP density samples. The following regression parameters were found

(1) $$\begin{array}{c} {\tilde{\rho }}_{0}\left(D\right)=\left[1.66\pm 0.02\right]-D\left[\left(5.1\pm 0.5\right)\times 10^{-5}\right], \end{array}$$

where ${\tilde{\rho }}_{0}$ defines a lateral density distribution within the upper sedimentary layer (in g/cm^3^) and *D* is the ocean depth (in m). The approximation of density samples by a higher-order regression model was found to be stochastically insignificant.

**Figure 4:**
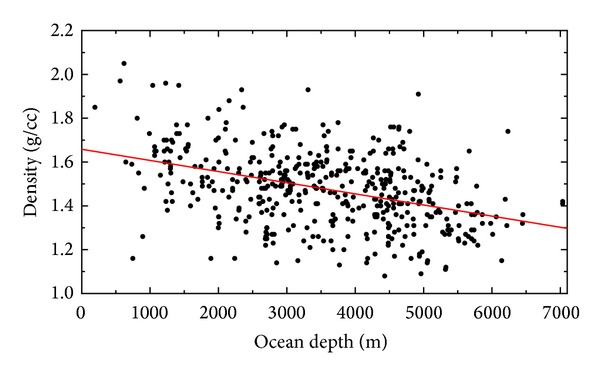
Relation between the marine sediment density and the ocean depth. The density samples were taken within the upper sediment layer (the sediment depth is <50 m). Theoretical density values (red line) were calculated using the upper sediment density equation in (1).

The upper sediment density equation in (1) defines the lateral density change as a function of the ocean depth. The estimated (nominal) sediment density of 1.66 g/cm^3^ is attributed to the upper sedimentary layer at sea level. The density proportionally decreases (with respect to this nominal value) at a rate of −0.051 g/cm^3^ per 1 km of the ocean depth (see Figure 4).

### 4.2. Depth Density Variation

**Figure 5:**
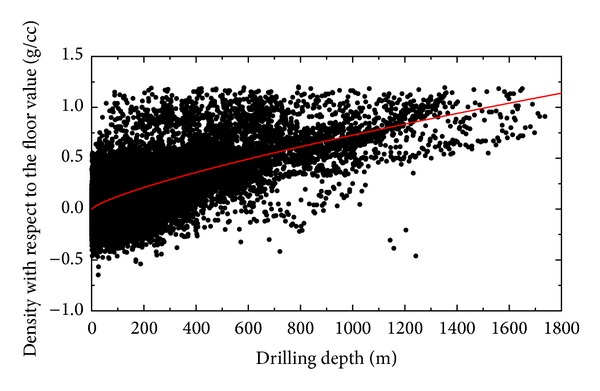
Relation between the (residual) marine sediment density and the sediment depth. The residual density values were calculated relative to a theoretical (upper layer) density ${\tilde{\rho }}_{0}$ (1). Theoretical density values (red line) were calculated using the expression in (2).

For the analysis of a depth-dependent density distribution, we first calculated the residual values for each drill site relative to a theoretical (upper layer) density of ${\tilde{\rho }}_{0}$ (see (1)). These residual density values were then fitted using the following power function:

(2) $$\begin{array}{c} \tilde{\delta }\rho \left(d_{\text{s}}\right)=\rho \left(d_{\text{s}}\right)-{\tilde{\rho }}_{0}\left(D\right)=\left[0.0037\pm 0.0002\right]d_{\text{s}}^{\left[0.766\;\pm \;0.007\right]}, \end{array}$$

where *d*_s_ is the drilling (sediment) depth (in m). The relation between the residual sediment density and the sediment depth is plotted in Figure 5. The density increases towards deeper stratigraphic units. The density-depth gradient has a decreasing tendency with the increasing sediment depth.

The density change with the sediment depth can also be fitted by the following logarithmic function:

(3) $$\begin{array}{c} \tilde{\delta }\rho \left(d_{\text{s}}\right)=\left[0.74\pm 0.03\right]\ln\left(1.0+d_{\text{s}}\left[0.00163\pm 0.00009\right]\right). \end{array}$$

As will be shown in Section 5, the power function in (2) provides a slightly better fit (by means of a standard deviation of the least-squares residuals between the measured and predicted density values) than the logarithmic function in (3). Since, the density-depth gradient attenuates with the increasing sediment depth, the exponential function is not appropriate for the approximation.

### 4.3. 3D Density Model

Combining (1) and (2), the 3D sediment density model is found to be

(4) ρ~(ds,D)=[1.66±0.02]−D[(5.1±0.5)×10−5] +[0.0037±0.0002]ds[0.766 ± 0.007].

Alternatively, the 3D sediment density model can be obtained from (1) and (3) in the following form:

(5) ρ~(ds,D)=[1.66±0.02]−D[(5.1±0.5)×10−5] +[0.74±0.03]ln⁡(1.0+ds[0.00163±0.00009]).

## 5. Model Uncertainties

**Figure 6:**
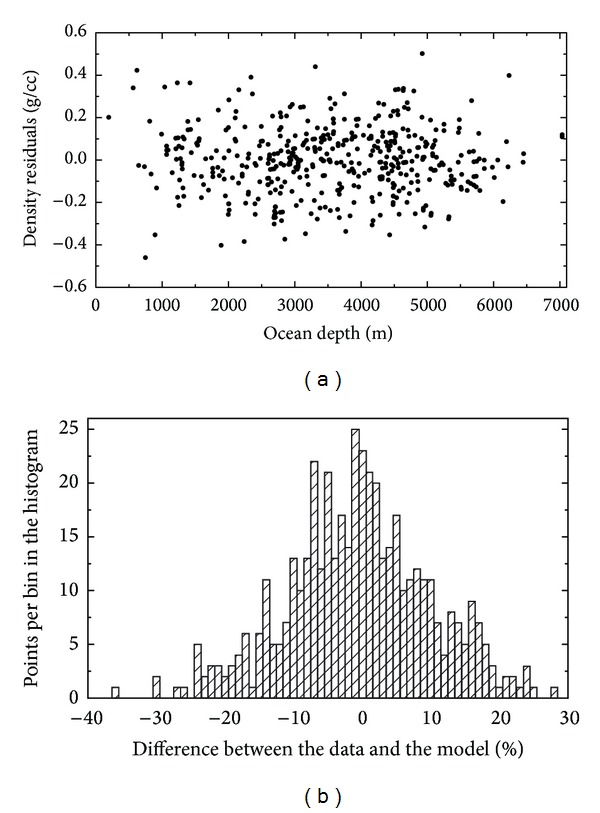
Errors of the upper sediment density equation in (1). Scatter plot and histogram of the differences between the measured and theoretical density values within the upper sedimentary layer (*d*_*s*_ < 50 m). The density differences are plotted with respect to the oceanic depth. Statistics of the differences: standard deviation = 0.16 g/cm^3^, max = 0.50 g/cm^3^, and min = −0.46 g/cm^3^. The histogram shows a relative distribution of the density differences.

The upper sediment density equation in (1) approximates the lateral density distribution within the upper sedimentary layer with an average error of 8.2%. This average error was computed as $\overset{-}{\varepsilon }=I^{-1}\sum_{i=1}^{I}\varepsilon_{i}$; $\varepsilon_{i}=\left(\left|\rho -\tilde{\rho }\right|/\tilde{\rho }\right)\times 100\%$, where *ρ* and $\tilde{\rho }$ are measured and theoretical density values, respectively. The uncertainties of the upper sediment density equation (1) are presented in Figure 6. The scatter plot shows differences between the measured and theoretical density values. The histogram shows distribution of relative density differences (in %). The density distribution exhibited clearly a decreasing trend with the increasing oceanic depth. The upper sediment densities are, however, also significantly dispersed (roughly ±0.4 g/cm^3^) around this prevailing trend.

**Figure 7:**
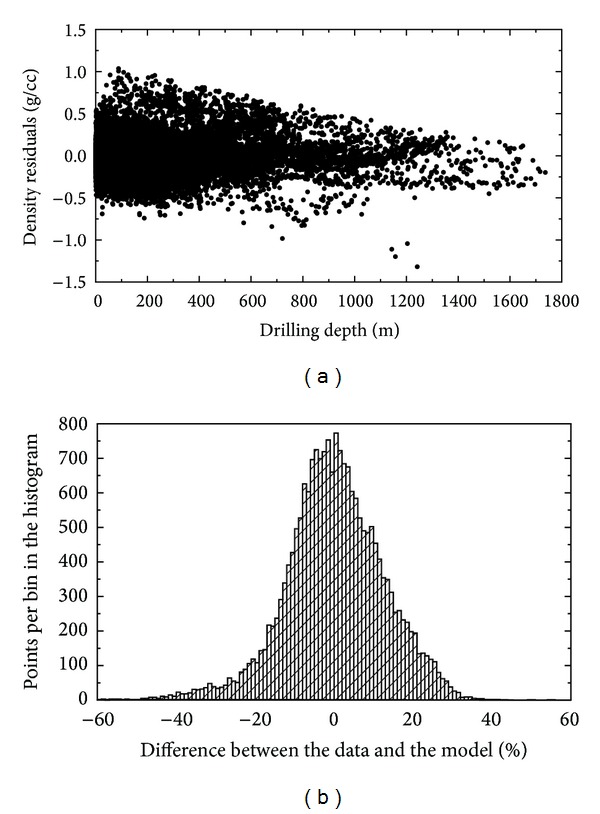
Errors of the 3D sediment density model in (3). Scatter plot and histogram of the differences between the measured and theoretical density values. The density differences are plotted with respect to the sediment depth. Statistics of the differences: standard deviation = 0.22 g/cm^3^, max = 1.04 g/cm^3^, and min = −1.32 g/cm^3^. The histogram shows the relative distribution of the density differences.

The 3D sediment density model in (4) approximates the NGDC density samples with an average error of 9.64%. The density model in (5) has slightly larger average error of 9.67%. The largest relative differences between the 3D density functions in (4) and (5) reach only 1.5%. These differences are about one order of magnitude smaller than average uncertainties of both density functions. Both models thus approximate the NGDC density samples with almost the same accuracy. The uncertainties of the 3D sediment density model (4) are shown in Figure 7. The largest density dispersions are within the upper sedimentary layers (mostly between −0.6 and 1.0 g/cm^3^). This dispersion is reduced at deeper sections (to roughly ±0.4 g/cm^3^ at the sediment depths below 1.25 km). We explain this by a more consolidated structure of deep sediments caused by compaction.

The theoretical density model in (1)–(5) that we derived under the assumption that the maximum density of marine sediments is 2.6 g/cm^3^. Moreover, the density samples within the 50 m thick upper sedimentary layer were used to establish the upper sediment density equation (1). For the maximum sediment density between 2.55 and 2.7 g/cm^3^, the parameter changes of both theoretical models in (4) and (5) are within the accuracy limits as found for the adopted maximum sediment density of 2.6 g/cm^3^. Moreover, the change of regression parameters in (1) is within the accuracy limits even if the density samples were taken within the upper sedimentary layer of which thickness is 100 m.

**Figure 8:**
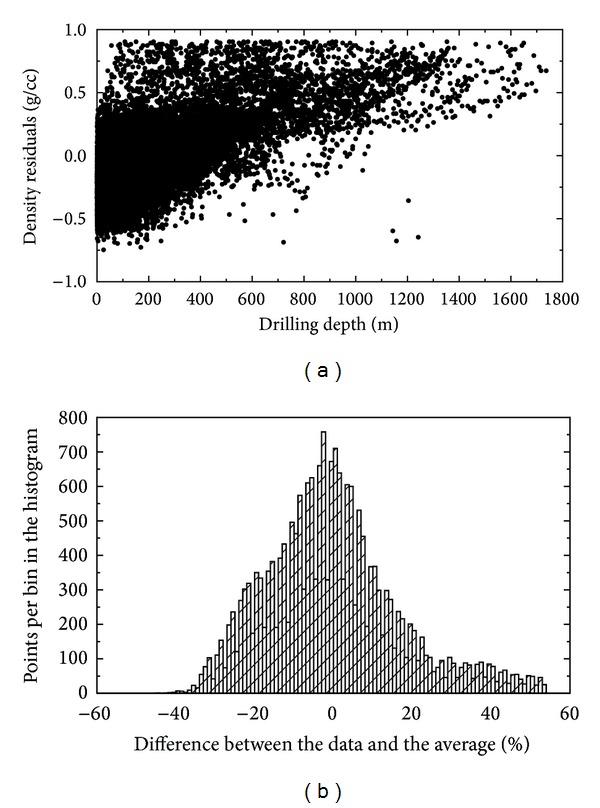
Errors of a uniform density distribution model. Scatter plot and histogram of the differences between the measured densities and the average sediment density of 1.70 g/cm^3^. Statistics of the differences: standard deviation = 0.29 g/cm^3^, max = 0.90 g/cm^3^, and min = −0.75 g/cm^3^. The histogram shows the relative distribution of density differences.

We further investigated the approximation errors of applying a uniform density model (for the average sediment density of 1.70 g/cm^3^). The scatter plot and histogram of errors are shown in Figure 8. The approximation of the NGDC density samples by a uniform density model yields an average error of 13.2%. The largest density dispersion (between −0.75 and 0.9 g/cm^3^) is seen in the upper stratigraphic units. This dispersion systematically decreases with the increasing sediment depth. As already explained, this pattern is likely due to a more consolidated structure of deep sediments. However, the application of a uniform density model systematically underestimates the sediment densities at deeper sections.

## 6. Remarks on Sediment Density Contrasts

The theoretical density models of marine sediments in (1) and (4) are utilized in definitions of the density contrasts of the ocean-sediment and sediment-bedrock interfaces. For the sediment-bedrock density contrast, we used the 3D sediment density model (4) and the average density of the DSDP bedrock density samples of 2.9 g/cm^3^. This value of the average bedrock density is slightly larger than the value given by Carlson and Raskin [50]. They estimated the average density of the oceanic crust of 2.89 ± 0.04 g/cm^3^ based on seismic refraction data in combination with drilling results and laboratory studies of seismic properties of oceanic and ophiolitic rocks and ophiolite lithostratigraphy. For the average bedrock density ${\overset{-}{\rho }}_{\text{b}}$ of 2.9 g/cm^3^, the sediment-bedrock density contrast Δ*ρ*_s/b_ is defined as

(6) Δρs/b(D,Ts)=ρ−b−ρ~(ds,D)≅[1.24±0.04]+[(5.1±0.5)×10−5]D −[0.0037±0.0002]Ts[0.766 ± 0.007],

where *T*_s_ is the total thickness of marine sediments (in m). With reference to an uncertainty of the average bedrock density as given by Carlson and Raskin [50], the expected uncertainty of the (nominal) sediment-bedrock density contrast at the shallow oceanic depths (i.e., in the first constituent on the right-hand side of (6)) is roughly ±0.04 g/cm^3^.

Gladkikh and Tenzer [51] developed a 3D seawater density model based on the analysis of oceanographic data from the World Ocean Atlas 2009 [52] and the World Ocean Circulation Experiment 2004 [53]. This 3D density model was defined as a function of the ocean depth (to account for density variations due to pressure) and geographical latitude (to account for density variations due to salinity and temperature). They also derived a more complex functional density model in order to account for a large seawater density gradient within the pycnocline, caused mainly by a combination of decreasing water temperature and increasing salinity with the increasing ocean depth. They estimated that this theoretical model approximates the actual seawater density distribution with a maximum relative error of 0.6%, while the corresponding average error is approximately 0.1%. These approximation errors of the seawater density model are considerably smaller than the estimated uncertainties of the upper sediment density equation in (1). The ocean-sediment density contrast can then be defined based on using a more simplified version of the seawater density-depth equation [54]

(7) $$\begin{array}{c} {\tilde{\rho }}_{\text{w}}\left(D\right)={\tilde{\rho }}_{\text{w},0}+\beta \left[a_{1}D+a_{2}D^{2}\right], \end{array}$$

where ${\tilde{\rho }}_{\text{w},0}=1.02791$ g/cm^3^, *β* = 6.37 g/m^3^, *a*_1_ = 0.7595 m^−1^_,_ and *a*_2_ = −4.3984 × 10^−6^ m^−2^.

From (1) and (7), the ocean-sediment density contrast Δ*ρ*_w/s_ is given by

(8) Δρw/s(ds,D)=ρ~0(D)−ρ~w(D)=[0.63±0.02]−[(5.6±0.5)×10−5]D +[(2.8±0.2)×10−11]D2.

The uncertainties in the sediment density model (1) represent most of the contribution to a total error budget of (8).

## 7. Discussion

**Figure 9:**
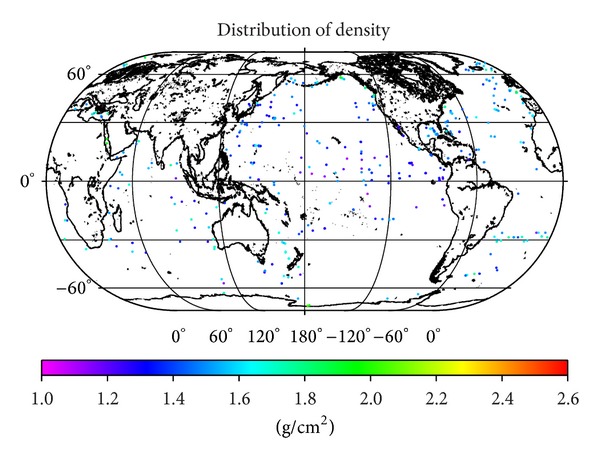
Values of the upper sediment density [in g/cm^3^] at 457 DSDP drill sites used for the analysis of the lateral density change with respect to the ocean depth.

The mineral composition and physical characteristics of marine sediments are the result of a complex interaction among geological, oceanographic, and biological processes (e.g., [55–57]). The transportation distance, the depositional environment conditions (depth, temperature, concentrations of dissolved gas, calcium carbonate, and silica), and the ocean fertility control both the sediment structure and the sedimentation process. Among these factors, the lateral density distribution depends primarily on mineral composition and transportation distance. Light and fine particles are transported at longer distances. Consequently, there is a clear pattern in the size distribution, for instance, of lithogenous sediments in the oceans (forming ~70% of total volume of marine sediments). Coarse particles (gravels and sands) form mostly near-shore deposits, while the grain size typically decreases offshore with clays occupying the deep-ocean basins. This might explain a prevailing trend of a decreasing (upper layer) sediment density with the increasing ocean depth (see Figure 4). To illustrate the dependence of the sediment density on the transportation distance, we compiled the map of upper density values taken at 457 DSDP drill sites in Figure 9. As seen, the upper sediment density at the bottom of marginal seas is typically higher than the density taken from drill sites at the bottom of deep oceans. The density minima are located throughout the equatorial Pacific, while the density maxima are at the bottom of the Red Sea.

Despite the fact that each stratigraphic unit exhibits a range of densities, the mean sediment density clearly increases with the increasing sediment depth. As seen in Figure 5, the density-depth gradient is slightly larger in shallow stratigraphic units than in deeper sediment sections. It is a well-known fact that this increasing sediment density is caused by compaction (cf. [27]). The increasing sediment density due to compaction causes the shallow sediment deposits in contact with the basement along the basin margins to have typically a lower density. The density contrast between the sediment-bedrock interface becomes less pronounced beneath deep sedimentary basins than along the margins of sedimentary basins as well as under shallow sediment accumulations. This has obviously implications on the accuracy of seismic and gravity surveys of deep sediment basins caused by a weakening signature of the basement interface with the increasing sediment depth and consequently the decreasing density contrast.

The 3D sediment density model in (4) approximates the NGDC density samples with an average error of 9.64%. A very similar accuracy is attained when using the alternative 3D density model in (5). On the other hand, the approximation by a uniform density model yields an average error of 13.2%. Despite the average errors, both models do not differ significantly; the 3D density model improved considerably the approximation of the density distribution at the greater sediment depths (>1 km). The 3D density model thus should be applied to represent the density structure of thick sediment deposits mostly found at the bottom of marginal seas.

Theoretical models of density and density contrast derived in this study can routinely be applied in global marine sediment studies. Moreover, these density models can be applied in regional studies, where sediment density samples are not available. As was demonstrated in Figure 1, the currently available NGDC database comprises the sediment density samples taken from only several hundreds of irregularly distributed drill sites. Most of the NGDC density samples were collected at the northern hemisphere except for the Arctic Ocean, where drill sites are almost completely absent. In regional studies with the known structure and physical properties of marine sediments, more customized models could be applied, adapting the existing density models (such as Hamilton's sediment density-depth equation), which were derived from a particular sediment type and its physical properties.

## 8. Summary and Concluding Remarks

We have derived the theoretical density models of marine sediments based on the analysis of the NGDC density samples. These density models were then utilized in deriving the theoretical models of the density contrast of the ocean-sediment and sediment-bedrock interfaces. The accuracy of the 3D sediment density model was analyzed and compared with a uniform density model.

The error analysis revealed that the upper sediment density equation in (1) approximates the lateral density variations with the average error of 8.2%. This equation together with the seawater density-depth equation in (7) was used for a definition of the ocean-sediment density contrast in (8). The density dispersion within the upper sedimentary unit is within ±0.4 g/cm^3^. The uncertainties of the seawater density model are, on the other hand, only about ±0.02 g/cm^3^. The uncertainties of the ocean-sediment density contrast are thus mostly attributed to the errors of the upper sediment density model.

The application of a uniform density distribution model yields large errors at deep marine sediment sections (>1 km) where it systematically underestimates the actual sediment density. Consequently, it overestimates the density contrast at the sediment-bedrock interface. The 3D sediment density models (in (4) and (5)) approximate more realistically the depth-dependent density change due to compaction. The average error of the 3D sediment density model in (4) was found to be 9.64%, while the average error of a uniform density model (of average sediment density of 2.7 g/cm^3^) is 13.2%. The 3D sediment density model also provides a more accurate representation of the sediment-bedrock density contrast (in (6)) than a uniform density model especially beneath thick sedimentary basins.
